# Tibial-graft fixation methods on anterior cruciate ligament reconstructions: a literature review

**DOI:** 10.1186/s43019-021-00089-0

**Published:** 2021-03-01

**Authors:** Vitor Luis Pereira, João Victor Medeiros, Gilvan Rodrigues Silva Nunes, Gabriel Taniguti de Oliveira, Alexandre Pedro Nicolini

**Affiliations:** 1 Traumatology Sports Center (CETE) – (DOT-UNIFESP/EPM) – Orthopedics and Traumatology Department of the Escola Paulista de Medicina, Federal University of São Paulo, Säo Paolo, Brazil; 2 Resident in the Orthopedics and Traumatology Program, Orthopedics and Traumatology Department of the Escola Paulista de Medicina, Federal University of São Paulo, Säo Paolo, Brazil

**Keywords:** Anterior cruciate ligament, Knee, Surgery, Review, Ligaments, Arthroscopy

## Abstract

**Introduction:**

Anterior cruciate ligament (ACL) reconstruction is the most performed orthopedic surgical procedure. The result of ACL reconstructions depends on multiple technical variables, including tension to be applied to the graft for fixation, knee-flexion angle during fixation and the type of fixation to the bone.

**Objective:**

To carry out a survey of the literature with the best evidence on these themes.

**Methods:**

Literature review about methods of tibial-graft fixation in ACL reconstructions – tension applied at the time of fixation, type of graft fixation, and knee-flexion degree during tibial fixation.

**Results:**

Thirty studies on the selected topics were found. Most studies point to graft-tension levels close to 90 N to obtain the best results. Regarding the knee-flexion angle, multiple studies suggest that fixation at a 30° angle would bring superior biomechanical advantages. Regarding the type of implant for fixation, it is not possible to affirm the superiority of one method over another in clinical outcomes.

**Conclusions:**

There is no consensus on the best method for tibial fixation of the grafts in ACL reconstructions regarding tension, type of implant and knee-flexion angle. However, the analysis of the studies pointed to certain trends and allowed the drawing of specific conclusions.

## Background

Anterior cruciate ligament (ACL) reconstruction is the most performed orthopedic surgical procedure [1, 2]. An ACL lesion is the most common ligamentous injury to the knee and results in 129,000 to 200,000 reconstructions per year in the United States and 400,000 worldwide [3].

Several reconstruction techniques have been described [4] including, especially, the isometric and the anatomical techniques. However, ACL anatomical reconstruction has been related to a better restoration of knee stability, both anteroposterior and rotational [5–7]. It is vital that surgeons understand the relationship between the tunnel position, graft fixation and graft length during knee-flexion-extension [8–10].

Several studies report that ACL reconstructions depends on multiple variables, including graft selection, intra-articular graft position, type of bone fixation, graft tension and knee-flexion angle at the time of fixation [11], with the last two surgical parameters under direct control of the surgeon. The association of these factors and the wide variety of fixation devices available allows the surgeon to directly influence knee kinematics and tibiofemoral compression forces [7, 12, 13]. The ideal amount of tension to be applied and the ideal knee-flexion angle at the time of tibial-graft fixation are still undetermined [14].

There are few studies on the topic in the literature, and the decision on which technique to use is often not based on objective criteria. Given this reality, we intend to present, compare and discuss the data and information found in the current literature on the best evidence levels available.

## Methods

This literature review was carried out from July to December 2019 in order to raise the best evidence of the theme found in the literature so far.

Our search strategy involved the terms “Ligament reconstruction” AND “Anterior Cruciate Ligament” AND “Tibial fixation” OR/AND “Surgical technique.” We use SCIELO, SCOPUS and PubMed as search platforms. In addition, referenced and manual searches were performed using Google Scholar and the platforms already mentioned.

Three independent authors selected and evaluated potential studies for inclusion in this review. Disagreements about the studies were solved through discussions between two authors and, when necessary, with the intervention of a third author.

**Fig. 1:**
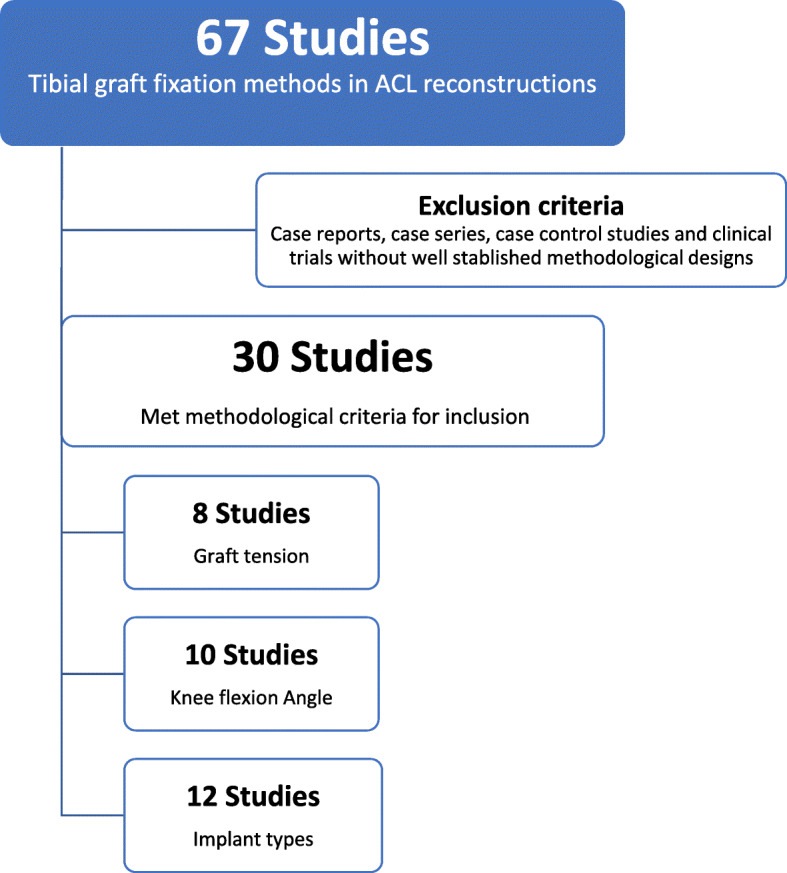
Flowchart showing the study selection method and characterization

All studies involving tibial-graft fixation methods in ACL reconstructions that fulfill adequate criteria for methodology and conduction were included. These criteria were based on the presence of an experimental design (clinical trials, randomized or not) and type of observation (case-control studies, cohort studies) performed on humans or cadaveric models (biomechanical studies), in addition to literature reviews, systematic reviews and meta-analyses presenting analytical evaluation of outcomes were included. Of a total of 67 studies found, 30 met the methodological criteria for inclusion (Fig. 1).

## Results

We identified and analyzed a total of 30 studies addressing the topic. There are multiple aspects associated with tibial-graft fixation during ACL reconstruction, highlighting three directly related variables: tension given to the graft, knee angle during fixation and implant types. Table 1 shows the characteristics of the works included in this study and their respective results for each of the highlighted topics.

**Table 1:** Included studies’ characteristics separated by theme. All the conclusions described on the table are based on a *p* value < 0.05

Author	Study type	***N***	Results
**Regarding the graft tension**			
Yasuda et al. [11] (1997)	Clinical trial	70	Initial relative high stress in the graft (about 80 N) decreases laxity
Bylski-Austrow et al. [12] (1990)	Experimental (biomechanical)	6	Stress magnitude is less influencing than fixation angle; there is no correct position or tension
Gertel et al. [14] (1993)	Experimental (biomechanical)	10	Graft strength and joint mobility unchanged by tension magnitude
Sherman et al. [1] (2012)	Review article	69	20–80 N of tension is recommended, depending on the graft, if flexion of 30°; 90 N of tension if in extension
Brady et al. [15] (2006)	Experimental (biomechanical)	12	Tension in extension generated greater compressive forces in the knee (90 N in extension = 3.5 x normal)
Austin et al. [16] (2007)	Experimental (biomechanical)	10	Graft tension did not change knee extension
Kim et al. [17] (2018)	Clinical trial	60	It is most appropriate to maintain a 20 -lb. (90 N) tension for graft fixation
Noyes et al. [18] (2019)	Experimental (biomechanical)		Current tensioning protocols are insufficient; suggests 40 flexion-extension cycles at 90 N for proper graft conditioning
**Regarding the knee-flexion angle when fixing the graft**			
Debandi et al. [5] (2016)	Experimental (biomechanical)	12	Anatomical reconstruction with fixation at 30° of knee flexion was superior (rotational stability)
Bylski-Austrow et al. [12] (1990)	Experimental (biomechanical)	6	Tension at 30° leads to greater stress in the graft than in extension; there is no correct position or tension
Gertel et al. [14] (1993)	Experimental (biomechanical)	10	Graft stress can be avoided with tensioning in extension
Brady et al. [15] (2006)	Experimental (biomechanical)	12	Stresses (15 N) applied at 20° of flexion or extension minimized rotational and axial forces on the knee; tension (90 N) in extension led to greater compressive forces
Austin et al. [16] (2007)	Experimental (biomechanical)	10	Knee flexion at 30° is associated with loss of extension
Mae et al. [19] (2008)	Experimental (biomechanical)	6	Knee flexion at 20° is closely associated to a normal knee
Kim et al. [17] (2018)	Clinical trial	60	Graft length shown to be longer with knee extended and loose in flexion
Miura et al. [20] (2006)	Experimental (biomechanical)	10	Dual band fixation (anteromedial/posterolateral bundles): PM bundle overloaded when fixed at 30°/30° and AM bundle overloaded when fixed at 60°/full extension
Höher et al. [21] (2001)	Experimental (biomechanical)	10	Fixing the graft at 30° of flexion better restored in situ forces and the kinematics of the knee when compared to the extension position
Asahina et al. [22] (1996)	Clinical trial	44	Superior stability and arthroscopic appearance in the group with the graft fixed at 30 ° of flexion; greater number of extension deficits when compared to fixation in extension
**Regarding the knee-implant types**			
Speziali et al. [23] (2014)	Systematic review	19	Clinical outcomes were good or excellent in 2/3 of patients regardless of implants
Steiner et al. [24] (1994)	Experimental (biomechanical)	36	If properly fixed, implants/tendons showed similar strength. Patellar tendon with interference screws showed increased rigidity
Scheffler et al. [25] (2002)	Experimental (biomechanical)	40	Bonding materials should be avoided. Use of bone block fixation or hybrid fixation may decrease chance of failure
Brand et al. [26] (2000)	Review article	98	Interference screws in bone-to-bone fixation seems superior; metallic and bioabsorbable screws with similar results
Eguchi et al. [27] (2014)	Experimental (biomechanical)	4	Fixed-length suspensory devices have a greater mechanical clamping force than those of an adjustable length
Benedetto et al. [28] (2000)	Clinical trial	113	Bioabsorbable polygluconate screws with similar results when compared to metallic screws
Drogset et al. [29] (2005)	Clinical trial	41	Metallic screws showed better results than bioabsorbable screws
Arama et al. [30] (2015)	Clinical trial	40	There are no clinical differences in the use of titanium screws and bioabsorbable screws with hydroxyapatite
Ma et al. [31]	Clinical trial	30	Fixation with interference screws shows no difference in outcomes when compared to suspensory fixation
Carulli et al. [32]	Clinical trial	90	Good and similar results when comparing combined fixation with interference screws/sheath versus interference screw/staple
Weiss et al. [33]	Experimental (biomechanical)	54	Hybrid fixation has biomechanical advantages over simple fixation
Teo et al. [34]	Clinical trial	64	Supplementary tibial-graft fixation did not benefit ACL reconstruction

Legend: *ACL* anterior cruciate ligament, *AM* anteromedial, *PM* posteromedial

### Regarding the graft tension

Eight studies were found addressing the theme. The amount of ideal force applied to the graft before fixation is a matter of considerable debate, with most authors recommending between 20 and 90 N of initial graft tension [1, 15].

### Regarding the knee-flexion angle when fixing the graft

Ten papers address the topic, with an angular variation described in the literature ranging from total extension to 30° of knee flexion [5]. There is no consensus on the most appropriate knee-flexion angle at the time of graft fixation, 30° being the knee-flexion angle that was the most recommended position in the majority of the studies [16].

### Regarding the implant types

Twelve papers were included in this analysis. It is known that no current implant or fixation method faithfully reproduces the native ACL fixation to bone surfaces [35]. In addition, the data available in the literature are highly conflicting, so that it is not possible to affirm the superiority of one method over another in clinical outcomes. The advantages and disadvantages of each method are multiple, as will be illustrated, and although the hybrid associations appear superior biomechanically, clinically this finding does not seem to translate into reality.

## Discussion

This revision pointed to certain trends and allowed the drawing of specific conclusions based on the results found by the studies’ analysis. Most authors recommended between 20 and 90 N of initial graft tension, knee flexion at 30° is the position most recommended by the majority of the studies and it is not possible to affirm the superiority of one fixation method over another in clinical outcomes.

### Regarding the graft tension

Some researchers suggested that tension measurements of a normal knee could be used as an intraoperative parameter in reconstructed knees [19]. However, complications to the knee position were observed in these protocols. The greatest compressive forces occurred when the graft was put under tension with the knee in extension, leading to significant external rotation and posterior translation of the tibia in relation to the femur [12, 15].

It was believed that undesirable changes in knee movement could occur if the graft were under excessive preload. In addition, the revascularization of autogenous grafts could be adversely affected [17]. Yasuda et al. [11] conducted a prospective clinical study with 70 patients undergoing ACL reconstruction, and concluded that the initial relatively high tension in the graft (about 80 N) decreases postoperative looseness in the knee.

Some older biomechanical studies have suggested that graft strength and joint movement would not be affected by the intraoperative tension magnitude. It was believed that some tension in the graft would be lost during fixation. If the magnitude of the tension had a minimal effect on joint mechanics, this loss of tension might not be an important factor in the result of the reconstruction [14, 15]. In contrast, more modern studies demonstrate progressive increases in graft elongation under a variety of cyclic loading conditions. Even so, there are problems in the applicability of these studies, as ACL grafts are cycled more than 500 times under high tension loads that would be difficult to perform during surgery. In addition, these studies do not test grafts in situ to determine the effect of the ACL graft and fixation devices on the knee kinematics. Consequently, no current graft-conditioning protocol based on objective criteria from a clinical point of view is accepted [18].

Noyes et al. [18] carried out a robotic biomechanical study on anatomical specimens to determine how to decrease postoperative graft elongation after ACL reconstruction. The study concluded that the current recommended protocols were not effective in preventing graft elongation after implant fixation and, therefore, risk the return of abnormal subluxations, proposing a more robust conditioning protocol of 40 cycles of extension and flexion during the application of an anterior tibial load of 90 N (20 lb). The data strongly suggested the need for increased load and graft cycling at the time of implantation to remove residual elongation after ACL reconstruction [18].

Excessive tensioning can also lead to problems, such as deficits in flexion. Kim et al. [17] measured changes in graft length to assess its intraoperative isometry in anatomical ACL reconstruction in vivo. They concluded that the single-bundle anatomical reconstruction of the ACL is not isometric, with the longest graft length in total extension and the loosest tension in flexion. The difference in the graft length changes between 20 (90 N) and 30 lb of tension was not statistically significant in knee-flexion angles below 90°. However, there was a significant difference in graft-length changes between the two tension groups in knee-flexion angles of 90° or more. This indicates that the graft should be secured using 20 lb (90 N) of tension instead of 30 lb to reduce the non-isometry degree.

### Regarding the knee-flexion angle when fixing the graft

Many authors argue that it is relatively easy to obtain a “tight” knee at 30° of flexion, in order to avoid residual instability. As the anteroposterior translation is greater near 30° [7] of knee flexion, an ACL replacement fixed in this position will become shorter than one fixed at any other flexion angle. This is probably the reason why some surgeons prefer this knee position during fixation. However, it is also easier to overconstrain the knee by tensioning at 30° [12].

Experimental data suggest that fiber recruitment and ACL tension increases as the knee moves from flexion to extension. If the graft fixation is performed at 20–30° of knee flexion and the fixation is not rigid, the graft may migrate proximally in the tibial bone tunnel or distal in the femoral tunnel during extension. If the ACL graft is rigidly fixed under high tension at 30° of knee flexion, over-constriction may occur, with loss of extension leading in the future to flexion contracture [15, 16].

The unidirectional collagen fibers of a reconstructed ACL usually do not reproduce the multidirectional fibers of a native ACL. If the unidirectional fibers are pulled tightly and the graft is fixed rigidly with the knee in flexion, loss of extension may occur. These concerns led surgeons to recommend ACL graft tensioning to be performed in full knee extension. In theory, graft tensioning and fixation in full extension should not result in loss of extension regardless of the amount of tension applied to the graft during fixation, preventing constriction of the knee [14, 16].

However, clinical and biomechanical studies have shown that a graft fixed at 30° better restores the stability of an intact knee than one fixed in full extension [5, 20]. Mae et al. [19] demonstrated that a graft fixed at 20° of knee flexion correlates better with the biomechanics of a normal knee, and is, therefore, the most desirable target. It is also worth considering that, according to Kim et al. [17] the length of the graft was longer with the knee fixed in extension, making it loose during flexion.

Höher et al. [21] evaluated the knee-flexion angle during graft fixation on a cadaveric study. They observed knee residual laxity in the total knee extension fixation when compared to 30° of flexion fixation, possibly explained by the increased tension applied to the graft when the knee was flexed, leading to greater stability. They concluded that 30° of flexion graft fixation better restored knee kinematics and in situ forces compared to an extension graft fixation.

Asahina et al. [22] conducted a comparative clinical study between ACL grafts fixed at 30° of flexion and grafts fixed in extension. They demonstrated that knee stability (using a KT-1000 arthrometer and the pivot-shift test) and arthroscopic appearance (volume, tension and synovial coverage) of the grafts were superior in the 30°-of-flexion group. However, they found a great number of extension deficits compared to the full extension graft fixation group.

Debandi et al. [5] conducted a biomechanical study to evaluate the effect of the knee-flexion angle on the hamstring graft fixation in full extension or at 30° of flexion. The tests were performed in both anatomical and non-anatomical reconstructions of the ACL. Anatomical reconstruction with graft fixation at 30° of flexion better restored the knee’s rotational stability. An ACL graft fixed in extension and in the anatomical position showed no difference when compared to non-anatomical ACL reconstructions. Therefore, a knee-flexion angle of 30° at graft fixation for ACL reconstruction should be considered to maximize the rotational stability of the knee.

### Regarding the implant types

The search for the development surgical techniques and new biomaterials capable of achieving ideal long-term results is a challenge. Current fixation devices have failed to reproduce the native ACL enthesis and the mechanical properties of the femur-ACL-tibia complex [35]. There are several objects of discussion and available graft-fixation devices [23, 36–38].

The graft-fixation methods in ACL reconstruction can be classified as suspension, post, compression or hybrid [24, 25]. Tibial fixation in ACL reconstruction is generally a point of less resistance than femoral fixation due to the lower density of the tibial bone and the parallel graft-fixation associated with the tunnel. This generates a sliding force that can cause early failure of the distal fixation [26].

Graft integration in the bone tunnel occurs around the 12th week [39], and early physical therapy rehabilitation is important for the clinical outcome of ACL reconstruction surgery. Therefore, secure fixation in the immediate postoperative period is essential to avoid displacement and impairment of the graft integration process [27].

Benedetto et al. [28] compared two methods of tibial-graft fixation: a bioabsorbable polygluconate screw and a metallic screw. One hundred and thirteen patients underwent ACL reconstruction, and were evaluated during a 1-year postoperative follow-up. The evaluations concluded that the results and the incidence of complications were similar. The study demonstrated that the polygluconate bioabsorbable screw is an effective alternative for ACL reconstruction.

Drogset et al. [29] evaluated 41 patients divided into two groups: 20 submitted to fixation with a metallic interference screw and 211 submitted to fixation with a bioabsorbable poly-L-lactic acid interference screw. In all reconstructions, patellar tendon grafts were used. Subjective knee functionality was better in patients who submitted to metal-screw fixation, showing less pain at rest, higher Tegner and Lysholm scores and better knee function after 2 years of follow-up. However, there was no significant difference in ligament stability.

Aiming to compare clinically and radiologically bioabsorbable interference screws with hydroxyapatite and titanium screw tibial-graft fixations, Arama et al. [30] developed a randomized clinical trial using hamstring grafts during a 5-year follow-up period. The study demonstrated equivalent clinical results between groups, 2 and 5 years after surgery. The bioabsorbable screw with hydroxyapatite provided adequate fixation and excellent functional results. There were no adverse effects. It was concluded that bioabsorbable screws with hydroxyapatite are a good alternative to titanium screws.

Ma et al. [31] compared three fixation methods for ACL reconstruction: a bioabsorbable interference screw, a suspensory fixation device and a post screw. Two groups of 15 patients underwent ACL reconstruction using hamstring autografts and were followed up for 2 years after surgery. The interference screw was used in both the femoral and tibial tunnels in one of the groups, while in the other the suspensory fixation device was used to fix the graft in the femoral tunnel, and the post screw for fixation in the tibial tunnel. All patients had a normal or close-to-normal International Knee Documentation Committee (IKDC) score. The results showed that rigid fixation with interference screws did not lead to significant differences in clinical outcomes when compared to suspensory fixation.

Carulli et al. [32] evaluated the clinical and radiographic outcomes between patients using the tibial resorbable screw and sheath versus the resorbable interference screw and staples. Ninety patients undergoing ACL reconstruction with hamstring grafts were randomized into two groups. Early and late complications were observed in both groups. In group B, there were symptoms associated with local intolerance to two metal clips, absent in group A. However, there were no failures related to fixation loss in either group.

Considering failure possibility related to the tibial fixation methods in ACL reconstruction, especially regarding the isolated interference screw, combined methods of fixation (hybrid fixation) have spread. Since then, several studies have been carried out to determine whether adding methods would effectively improve the initial stiffness of the system. Regarding this subject, there is a divergence between studies [24, 33].

Weiss et al. [33] performed a comparative biomechanical tibial fixation strength analysis for ligament reconstruction with interference screws, post screws with a pin and washer and both methods (hybrid fixation). The hybrid fixation group showed significantly higher final stiffness compared to the other groups (*p* < 0.05) and higher performance when compared to the interference screw group.

Teo et al. [34] conducted a study to assess whether additional staples for tibial fixation of the graft would be necessary in ACL reconstructions. A total of 64 patients were included in the study, being divided into two groups: the isolated bioabsorbable interference screw and additional fixation with a clamp. Both groups were evaluated after 1 year of follow-up. There was no statistically significant difference between the groups, concluding that the additional fixation of the tibial graft did not present additional benefits and was, therefore, not necessary during ACL reconstruction.

Such findings are impactful regarding their applicability to sports. The very frequent ACL injuries in athletes require effective and efficient treatment to maximize results and return to sports, giving priority to return at the same previous level. Another important matter refers to a quick and safe return, with the minimum recovery time without risking a new lesion. In this way, this paper can offer certain technical choices that are safe and maximize the surgeon’s trust in the tibial-graft fixation. Therefore, it is possible to prioritize the gain of performance, rehabilitation and high demand in these athletes without risking the surgical procedure.

## Conclusions

There is no literary consensus about the best tension to be applied. It is believed that too little tension can lead to residual laxity while too much tension can cause movement restriction. Most studies point to tension levels close to 90 N to obtain the best results.

Although, in fact, there is no well-established and no ideal method of fixation, and descriptions range from 30° of flexion to full extension, multiple studies suggest that fixation at an angle of 30° of knee flexion would bring superior biomechanical advantages in ACL function without angle-related significant complications.

Regarding the use of implants, the data found in the literature are highly conflicting, so that it is not possible to affirm the superiority of one method over another in clinical outcomes. The advantages and disadvantages of each method are multiple, and although the hybrid associations appear superior biomechanically, clinically this finding does not seem to translate into reality.

## Data Availability

Not applicable
